# Innate immune cell-intrinsic ketogenesis is dispensable for organismal metabolism and age-related inflammation

**DOI:** 10.1016/j.jbc.2023.103005

**Published:** 2023-02-10

**Authors:** Emily L. Goldberg, Anudari Letian, Tamara Dlugos, Claire Leveau, Vishwa Deep Dixit

**Affiliations:** 1 Department of Physiology, University of California San Francisco, San Francisco, California, USA; 2 Department of Pathology, Yale School of Medicine, New Haven, Connecticut, USA; 3 Department of Comparative Medicine, Yale School of Medicine; 4 Department of Immunobiology, Yale School of Medicine; 5 Yale Center for Research on Aging, Yale School of Medicine

**Keywords:** ketone, aging, innate immunity, inflammation, metabolism, BDH1, 3-hydroxybutyrate dehydrogenase 1, BHB, β-hydroxybutyrate, BMDM, bone marrow–derived macrophages, HMGCL, 3-Hydroxy-3-Methylglutaryl-CoA Lyase, HMG-CoA, hydroxy-β-methylglutaryl CoA, HMGCS2, 3-Hydroxy-3-Methylglutaryl-CoA Synthase 2, Kbhb, lysine β-hydroxybutyrlation, KD, ketogenic diet(s), LPS, lipopolysaccharide

## Abstract

Aging is accompanied by chronic low-grade inflammation, but the mechanisms that allow this to persist are not well understood. Ketone bodies are alternative fuels produced when glucose is limited and improve indicators of healthspan in aging mouse models. Moreover, the most abundant ketone body, β-hydroxybutyrate, inhibits the NLRP3 inflammasome in myeloid cells, a key potentiator of age-related inflammation. Given that myeloid cells express ketogenic machinery, we hypothesized this pathway may serve as a metabolic checkpoint of inflammation. To test this hypothesis, we conditionally ablated ketogenesis by disrupting expression of the terminal enzyme required for ketogenesis, 3-Hydroxy-3-Methylglutaryl-CoA Lyase (HMGCL). By deleting HMGCL in the liver, we validated the functional targeting and establish that the liver is the only organ that can produce the life-sustaining quantities of ketone bodies required for survival during fasting or ketogenic diet feeding. Conditional ablation of HMGCL in neutrophils and macrophages had modest effects on body weight and glucose tolerance in aging but worsened glucose homeostasis in myeloid cell-specific Hmgcl-deficient mice fed a high-fat diet. Our results suggest that during aging, liver-derived circulating ketone bodies might be more important for deactivating the NLRP3 inflammasome and controlling organismal metabolism.

Mammals have evolved to prioritize glucose for energy. A complex, carefully regulated system has developed to control glucose availability and utilization for every cell in the body. However, during periods of starvation or limited glucose availability, mammals break down fat, leading to the production of ketone bodies, to supply energetic demand (1). Ketone bodies are short chain fatty acids that fuel cellular ATP production through their ability to enter the TCA cycle. Thus, ketone bodies are often considered alternative metabolic fuels. Notably, many metabolic interventions that induce ketogenesis also extend lifespan in model organisms (2, 3, 4, 5). Moreover, ketogenic diets (KD) improve markers of healthspan in old mice (6, 7). Collectively, these studies underscore an important role for ketone bodies in aging and healthspan.

The metabolism of ketone bodies has been expertly reviewed previously (8). Free fatty acids liberated from adipose tissue through lipolysis are broken down through β-oxidation in the liver, leading to the production of acetyl-CoA. As the concentration of acetyl-CoA increases in hepatocyte mitochondria, it is converted to ketone bodies through a series of enzymatic reactions. The final step in ketogenesis is catalyzed by the enzyme 3-Hydroxy-3-methylglutaryl-CoA lyase (HMGCL, encoded by the gene *Hmgcl*) to form the ketone body acetoacetate, which can then be converted to the other ketone bodies β-hydroxybutyrate (BHB) and acetone. Hepatocytes do not express the enzyme required for ketolysis Succinyl-CoA:3-Ketoacid-CoA Transferase (encoded by the gene *Oxct1*), and this preserves ketone bodies for extrahepatic tissues like the brain, heart, and skeletal muscle (9).

In addition to this classical regulation of ketogenesis, recent evidence shows nonhepatic sources of ketone bodies impact a variety of organ systems. In adipose tissue, beige adipocytes secrete BHB that is oxidized by adipocyte precursors to preserve adipogenic differentiation and limit fibrotic lineage skewing (10, 11). BHB is also produced by small intestine stem cells and this is important for maintaining their stemness within crypts (12, 13). Local ketone production has been reported in CD8 T cells and implicated to regulate their memory response (14). Renal epithelial cells produce BHB to mediate protective effects of nicotinamide (15). The failing heart also increases ketone body consumption (16, 17, 18). Finally, macrophages can oxidize acetoacetate depending on their inflammatory state, and this is important for protecting against liver fibrosis (19, 20). Collectively, these studies emphasize that ketone bodies may have autocrine/paracrine functions and have broad physiological importance.

Ketone bodies are also pleiotropic signaling molecules. BHB acts as a histone deacetylase inhibitor to control gene expression (21). Similar to other short chain fatty acids, BHB can covalently bind lysine residues on histones and other proteins, although the importance of this posttranslational modification is not well understood (14, 22, 23, 24). In addition, we previously showed that BHB inhibits NLRP3 inflammasome activation in macrophages (25) and neutrophils (26). Persistent low-grade inflammation is believed to underlie many diseases of aging (27) and we have previously shown that the NLRP3 inflammasome is a key driver of age-related and obesity-driven inflammation (28, 29). Based on the broad actions of BHB, we hypothesized that ketone bodies might be an important regulatory checkpoint for chronic inflammation in aging.

Ketone bodies impact a wide range of immune functions (14, 19, 25, 26, 30, 31, 32, 33, 34, 35, 36). While several studies have investigated the fate of extracellular ketone bodies in immune function, less is known about immune cell-intrinsic ketogenesis. To test the importance of ketone body production in macrophages and neutrophils, we developed a novel mouse model by targeting *Hmgcl (Hmgcl*^*fl/fl*^*)* to conditionally ablate ketone body synthesis in specific cell types. This strategy allowed us to focus exclusively on ketone body production, in contrast to preexisting models targeting the upstream rate-limiting enzyme HMGCS2 (13) or the downstream 3-hydroxybutyrate dehydrogenase 1 (BDH1) that interconverts acetoacetate to BHB (16). By crossing these mice to liver-specific Albumin-Cre (Hmgcl^Alb-Cre^), we show that despite the presence of nonhepatic ketogenesis, the liver is the only organ that can produce enough ketone bodies to support survival under ketogenic conditions. We also find that neutrophil (using S100a8-Cre, Hmgcl^S100a8-Cre^)-intrinsic ketogenesis does not regulate age-related metabolic health. In addition, using LysM-Cre to ablate ketogenesis in all myeloid cells (Hmgcl^LysM-Cre^), we find only modest impacts on age- and obesity-induced metabolic dysregulation. These data suggest that innate immune inflammation is controlled by extracellular ketone bodies and that innate immune-intrinsic ketogenesis does not regulate age-related inflammation and metabolic health defects in aging.

## Results

**Figure 1:**
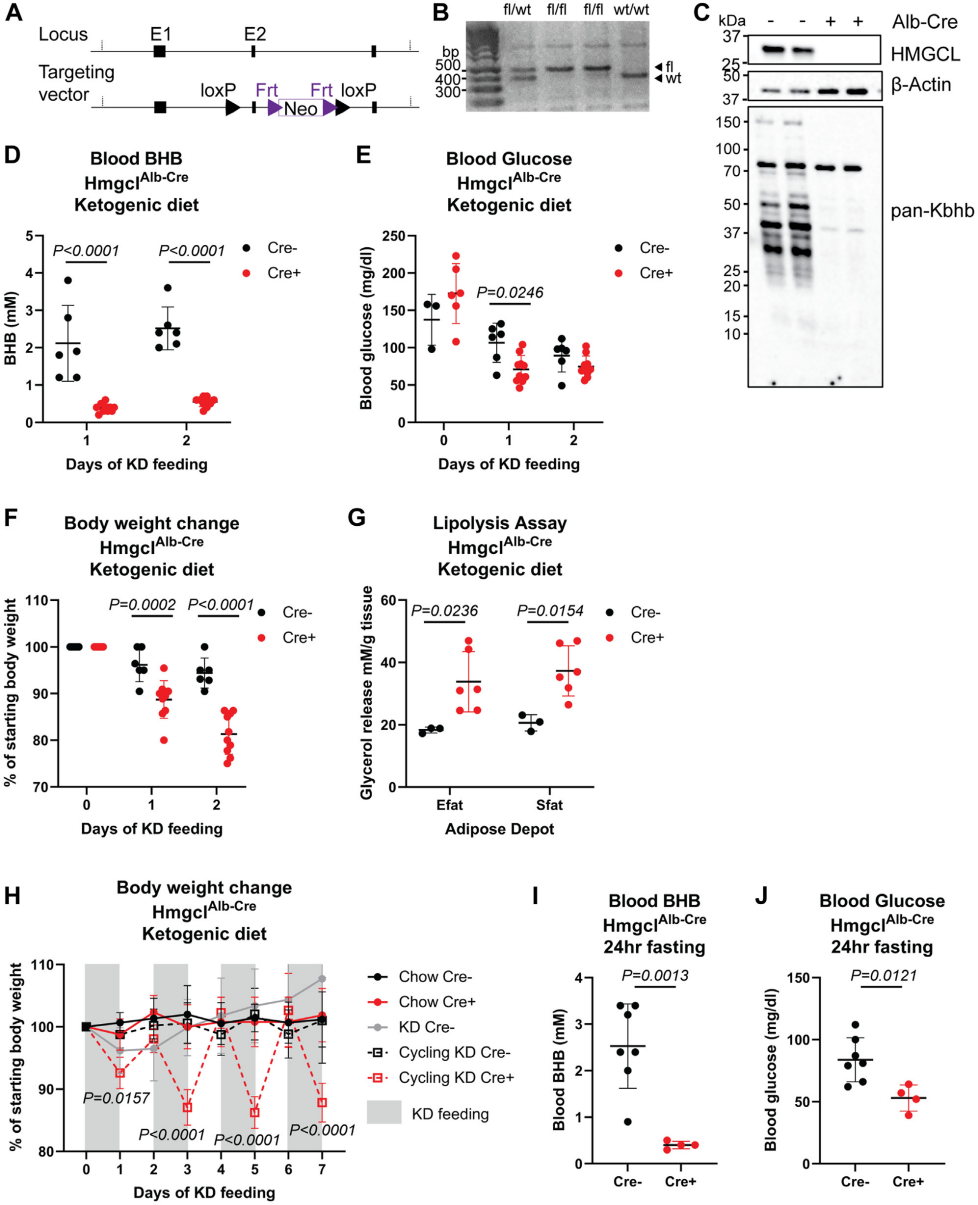
**Development and validation of a novel mouse model to conditionally ablate ketogenesis.** A mouse was generated to study cell-specific ketogenesis by targeting expression of *Hmgcl*. *A*, targeting vector design to introduce loxP sites to allow Cre-mediated excision of exon 2 within the *Hmgcl* gene. *B*, representative DNA genotyping gel. *C*, liver-specific *Hmgcl* ablated mice were fed a ketogenic diet for 1 week. Protein expression of HMGCL, Actin, and pan-Kbhb were assessed in livers of Cre+ and Cre- Hmgcl^Alb-Cre^ mice by Western blot. *D*, blood BHB, (*E*) blood glucose, and (*F*) body weights were measured in Hmgcl^Alb-Cre^ mice–fed KD each morning. *G*, glycerol release from Efat and Sfat was measured after 48 h of KD feeding. For (*D*–*G*), each symbol represents an individual mouse, and data are presented as mean ± SD, and all statistical differences were calculated by 2-way ANOVA to compare genotypes at each time point. *H*, body weights were measured each morning during 1 week of cycling KD feeding. Statistical differences between Cre+ and Cre- Hmgcl^Alb-Cre^ littermates were calculated by 2-way ANOVA. Data are represented as mean ± SD. *I*, blood BHB and (*J*) blood glucose levels were measured after 24 h fasting in Hmgcl^Alb-Cre^ mice. For *I* and *J*, each symbol represents an individual mouse and statistical differences were calculated by student’s *t* test. Data are represented as mean ± SD. BHB, β-hydroxybutyrate; HMGCL, 3-Hydroxy-3-Methylglutaryl-CoA Lyase; Kbhb, lysine β-hydroxybutyrlation; KD, ketogenic diet.

To test the role of ketogenesis within innate immune cells, we first generated a mouse model containing a loxP-flanked region of exon 2 within the *Hmgcl* gene and verified homozygosity in genomic DNA (Fig. 1, *A* and *B*). To validate functional gene targeting, we first crossed these Hmgcl^fl/fl^ mice with the liver-specific Albumin-Cre (Hmgcl^Alb-Cre^) and confirmed protein deletion (Fig. 1*C*). In contrast to whole-body HMGCL deficiency that is embryonic lethal (37), liver-specific Hmgcl-deficient mice fed a normal chow diet were viable. When fed a KD, Hmgcl^Alb-Cre^ mice failed to increase circulating blood BHB concentration (Fig. 1*D*) and Cre+ mice had lower blood glucose (Fig. 1*E*). These data agree with a prior study in an independent Hmgcl^fl/fl^ model that was published while we were developing our mice (38). Interestingly, when fed KD, the mice also fail to induce lysine β-hydroxybutyrlation (referred to as Kbhb) (Fig. 1*C*), a newly described posttranslational modification by BHB (22). Concomitant with their inability to induce hepatic ketogenesis, Hmgcl^Alb-Cre^ mice failed to maintain body weight during KD feeding (Fig. 1*F*), primarily due to increased adipose tissue lipolysis (Fig. 1*G*). Notably, the weight-loss phenotype could be rescued if we cycled mice off KD every 24 h in exchange for standard chow (Fig. 1*H*), demonstrating the specificity of HMGCL-mediated hepatic ketogenesis in maintaining body weight. In contrast to liver-specific BDH1 KO mice that still increase ketone body concentrations in response to fasting (39) and liver-specific PPARa KO mice that have lower but inducible ketone bodies in response during sepsis (40), Hmgcl^Alb-Cre^ mice also fail to induce ketogenesis in response to fasting (Fig. 1*I*) and they have lower blood glucose levels under fasting conditions (Fig. 1*J*). All together, our data functionally validate HMGCL ablation in this new mouse model and demonstrate the liver is the only organ that can supply enough ketone body production under ketogenic conditions and that no other tissues can compensate for hepatic ketogenesis to meet whole-body energy requirements during KD feeding or fasting.

**Figure 2:**
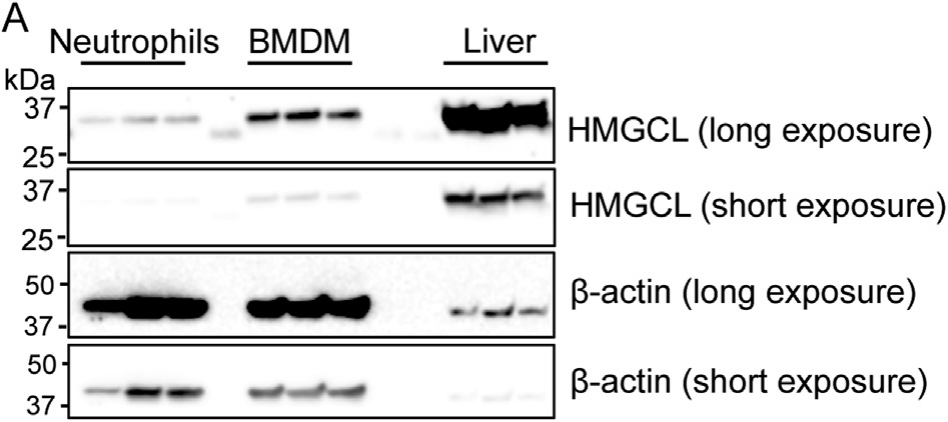
**Comparison of HMGCL expression between liver and myeloid cells.** HMGCL protein expression was compared in neutrophils, bone marrow-derived macrophages (BMDMs), and whole liver tissue by Western blot. Short and long exposures are provided as indicated. Each lane is an individual mouse. HMGCL, 3-Hydroxy-3-Methylglutaryl-CoA Lyase.

**Figure 3:**
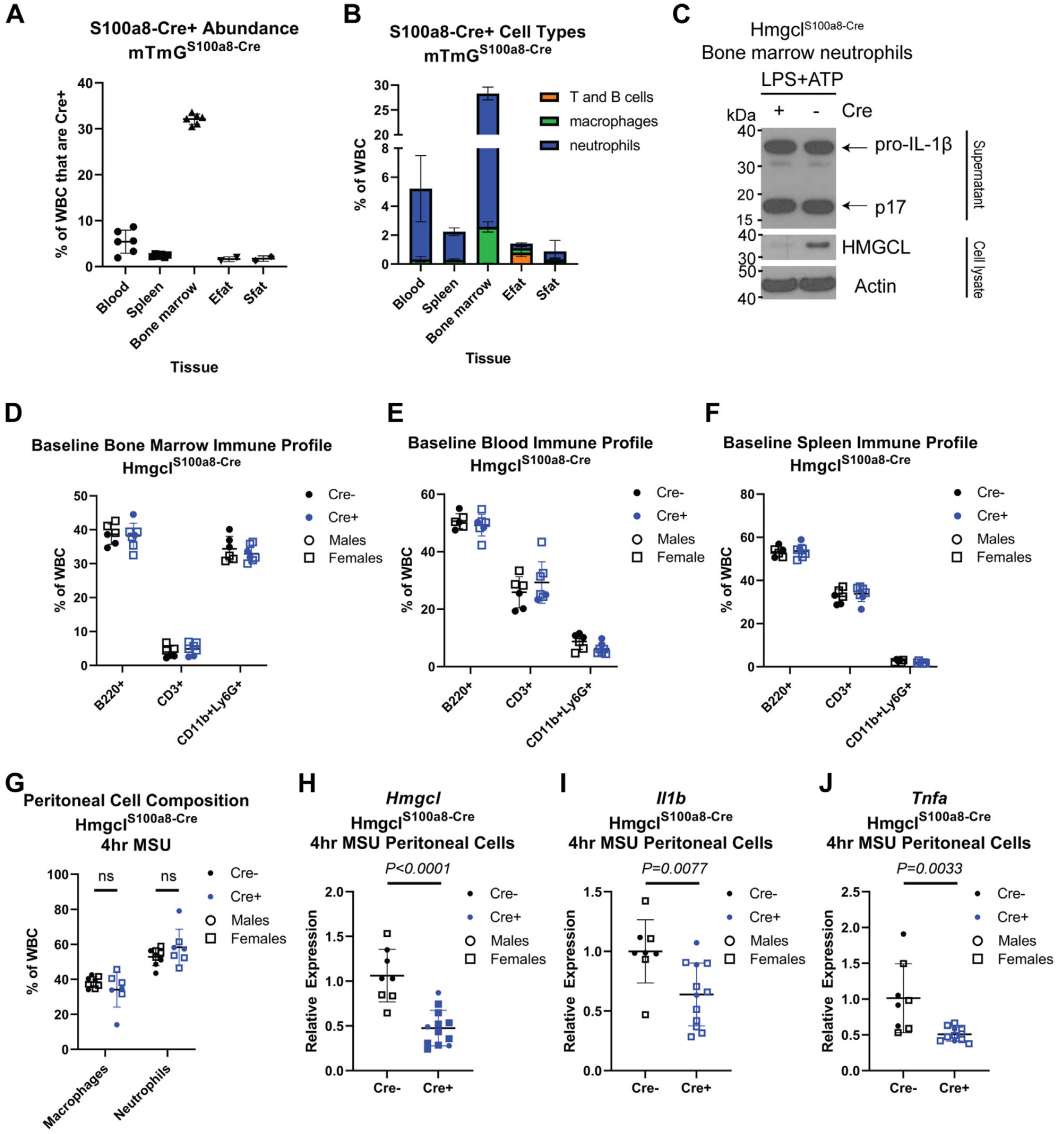
**Validation and baseline characterization of HMGCL ablation in neutrophils.** For (*A* and *B*), S100a8-Cre^+^ mice were crossed to mTmG reporter mice to verify neutrophil-specificity. *A*, the total abundance of Cre+ (mGFP+) cells in each tissue were measured by flow cytometry. Each symbol represents an individual animal except for Efat and Sfat, which are pooled n = 2 mice/symbol. *B*, within Cre+ cells in each tissue, the cell lineage composition was determined by flow cytometry. For both graphs, data are expressed as mean ± SD. *C*, representative western blots from stimulated neutrophils isolated from bone marrow of Hmgcl^S100a8-Cre^ mice after NLRP3 inflammasome activation with LPS + ATP. *Top panel* show IL-1β secretion in culture supernatants. Lower blots show HMGCL and Actin expression in cell lysates. For (*D*–*J*), Hmgcl^S100a8-Cre+/−^ mice were compared to Hmgcl^fl/fl^ littermates. Baseline B220+ B cells, CD3+ T cells, and neutrophils were assessed in (*D*) bone marrow, (*E*) blood, and (*F*) spleen. Inflammatory response was assessed in the total peritoneal cell exudate by measuring (*G*) macrophage and neutrophil infiltration and gene expression of (*H*) *Hmgcl*, (*I*) *Il1b*, and (*J*) *Tnfa* by RT-PCR. Male (*circles*) and female (*squares*) Cre+ (*black*) and Cre- (*blue*) littermates were combined for analysis and each symbol represents an individual mouse. Data are expressed as mean ± SD. For (*G*), statistical differences were calculated by 2-way ANOVA to test for differences between genotypes for macrophage or neutrophil differences. For (*H*–*J*), statistical differences were calculated by *t* test. HMGCL, 3-Hydroxy-3-Methylglutaryl-CoA Lyase; LPS, lipopolysaccharide.

Based on our prior findings that neutrophil NLRP3 inflammasome activation can be regulated by BHB and the unexpected expression of ketogenic enzymes in these short-lived glycolytic immune cells (Fig. 2), we tested the importance of neutrophil-intrinsic ketogenesis by crossing the Hmgcl^fl/fl^ mice to the neutrophil-specific S100a8-Cre (Hmgcl^S100a8-Cre^). Cre specificity was assessed by crossing to the mT/mG reporter mouse, mT/mG^S100a8-Cre^ (Fig. 3, *A* and *B*), that indelibly marks Cre-recombined cells with membrane GFP (mG). We induced NLRP3 inflammasome activation in isolated bone marrow neutrophils and found that HMGCL does not regulate NLRP3-dependent IL-1β secretion (Fig. 3*C*). The immune phenotyping of adult male and female mice revealed that HMGCL ablation in neutrophils does not impact peripheral neutrophil abundance nor does it have indirect effects on other lymphoid populations (Fig. 3, *D*–*F*). However, when we gave Hmgcl^S100a8-Cre^ mice intraperitoneal (ip) injections of monosodium urate crystals, the causative agent of gout and potent neutrophil stimulus, HMGCL-deficient neutrophils had similar migration into the peritoneal cavity, but there was an overall lower inflammatory response based on Il1b and Tnfa gene expression (Figs. 3, *G*–*J* and S1). These data confirm efficient deletion of HMGCL using S100a8-Cre that does not impact overall neutrophil abundance in the periphery, but HMGCL-deficient neutrophils have moderately lower inflammatory responses to certain stimuli.

**Figure 4:**
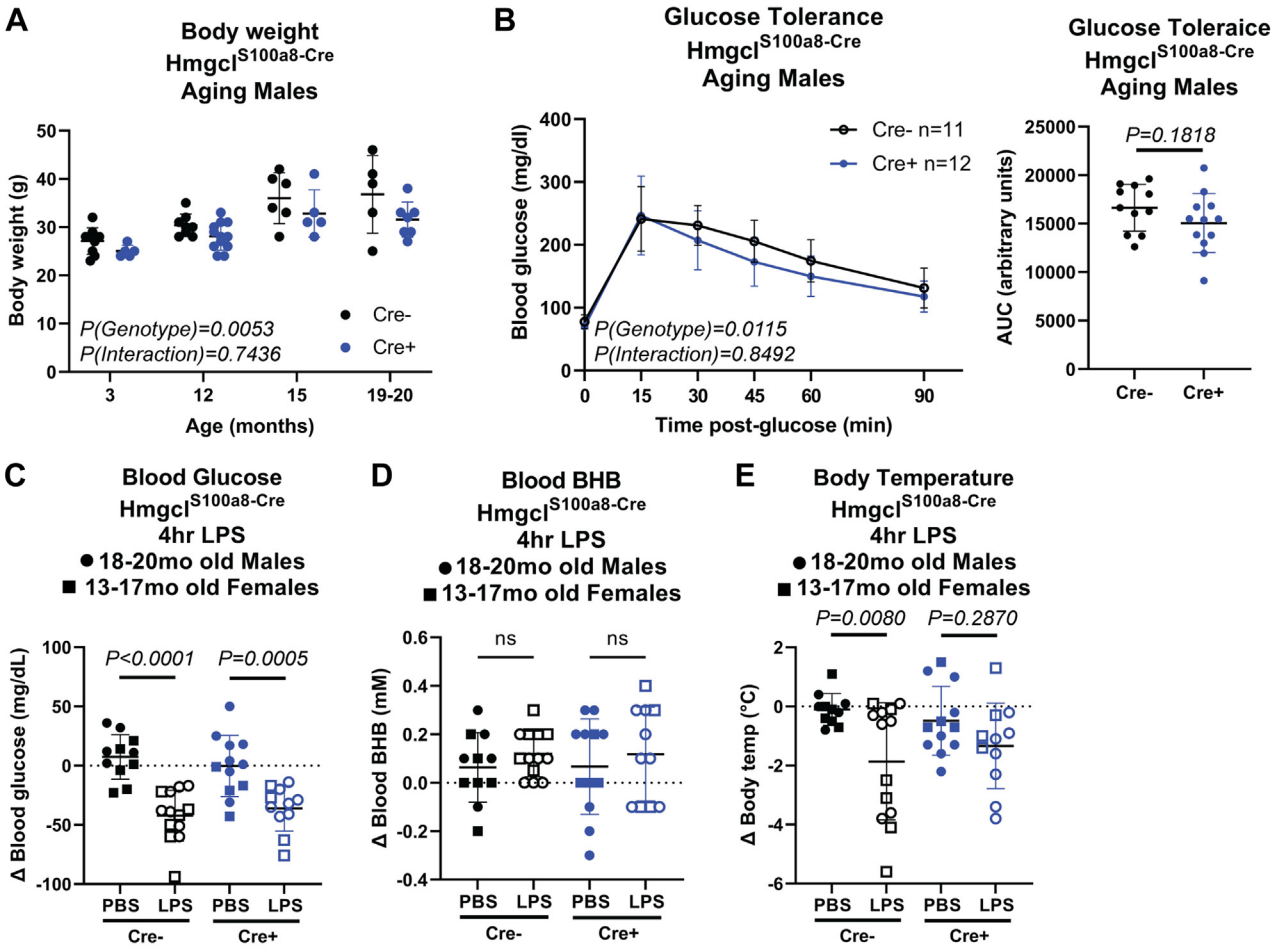
**Neutrophil-specific *Hmgcl* ablation does not impact age-related inflammation.** Hmgcl^S100a8-Cre^ male mice were aged to at least 18 months old. *A*, body weights of independent cohorts at varying ages. Statistical differences were calculated by 2-way ANOVA. *B*, glucose tolerance test of males aged 15 to 18 months old. Area under the curve was quantified for each animal (*right side panel*). Statistical differences were calculated by paired 2-way ANOVA (*left*) or *t* test (*right*). For (*C*–*E*), 18 to 20 month-old males (*circles*) and 13 to 17 month-old females (*squares*) were injected with LPS or PBS control and changes in (*C*) blood glucose, (*D*) blood BHB, and (*E*) body temperature were measured 4 h later to assess the physiological response to acute inflammation. Statistical differences in (*C*–*E*) were calculated by 1-way ANOVA with Sidak’s correction for multiple comparisons within each genotype (ns: not significant). For all graphs, each symbol represents an individual mouse and all data are expressed as mean ± SD. BHB, β-hydroxybutyrate; HMGCL, 3-Hydroxy-3-Methylglutaryl-CoA Lyase; LPS, lipopolysaccharide.

Upon aging, male Hmgcl^S100a8-Cre^ mice showed modest differences in body weights (Fig. 4*A*). Likewise, neutrophil-specific deletion of *Hmgcl* also modestly protected 15 to 20 month-old male mice from age-related glucose intolerance compared to male Cre-negative littermate controls (Fig. 4*B*). To test if HMGCL ablation altered acute inflammatory responses during aging, we analyzed physiological responses to intraperitoneal injection with gram-negative bacterial cell wall component lipopolysaccharide (LPS) that activates TLR4 signaling. While we measured expected changes in blood glucose and body temperature in LPS-challenged male and female mice, Cre+ mice did not have significantly lower body temperature after LPS injection, possibly due to using a low dose (Figs. 4, *C*–*E* and S2). Collectively, these data suggest that neutrophil-intrinsic HMGCL expression, and hence neutrophil-intrinsic ketogenesis, has modest impacts on age-related health indicators but is not a major regulator of inflammation during aging.

**Figure 5:**
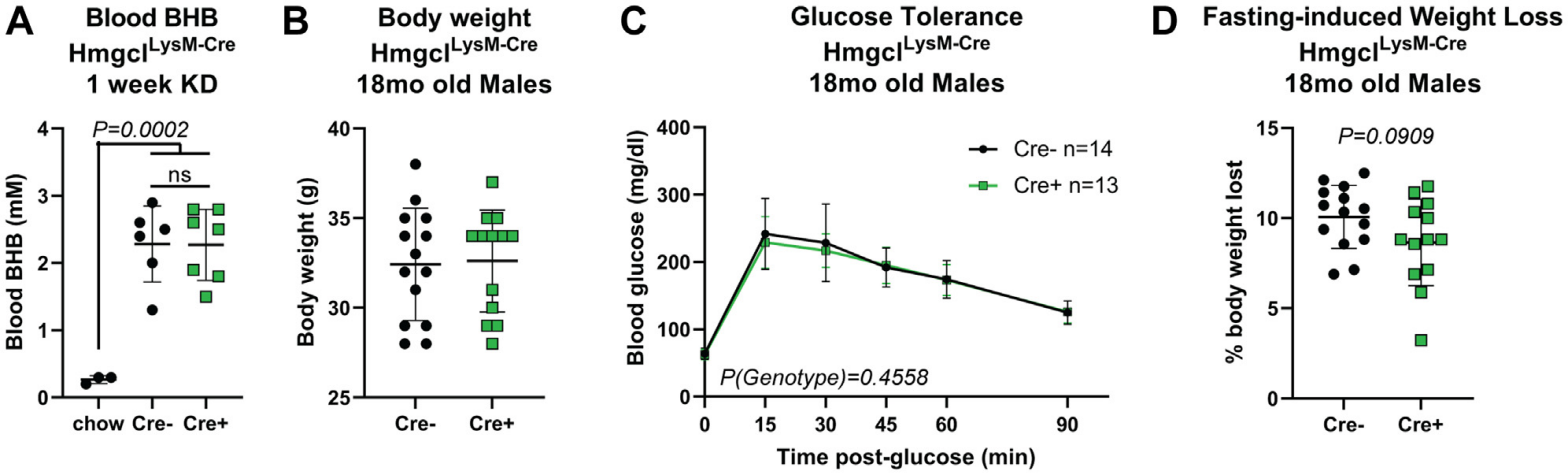
**Myeloid-specific *Hmgcl* expression does not regulate metabolic health during aging.***A*, Hmgcl^LysM-Cre^ were fed KD for 1 week to measure blood BHB levels. Statistical differences were calculated by 1-way ANOVA with Tukey’s correction for multiple comparisons. Each symbol represents an individual mouse and data are represented as mean ± SD. For (*B*–*D*), Hmgcl^LysM-Cre^ male mice were aged to 18 months old and assessed for basic metabolic health parameters compared to their Cre-negative Hmgcl^fl/fl^ littermates. *B*, body weights, (*C*) glucose tolerance, and (*D*) 16-h fasting-induced weight loss were measured. For all graphs, each symbol represents an individual mouse. For *B* and *D*, statistical differences were calculated by unpaired student’s *t* test between Cre- and Cre+ groups. For *C*, each mouse was individually tracked, so statistical differences were calculated by paired 2-way ANOVA. Data are expressed as mean ± SD. BHB, β-hydroxybutyrate; HMGCL, 3-Hydroxy-3-Methylglutaryl-CoA Lyase; KD, ketogenic diet.

Because macrophage function is also regulated by ketone bodies (19, 25), we broadened our scope by assessing the role of HMGCL in all myeloid-lineage cells. For these experiments, we crossed Hmgcl^fl/fl^ mice with mice expressing the generic myeloid LysM-Cre driver (Hmgcl^LysM-Cre^) to ablate HMGCL in all myeloid cells *in vivo*. After 1 week of KD feeding, Cre-positive and Cre-negative adult littermates had similar blood BHB levels (Fig. 5*A*), confirming that myeloid cells do not contribute to whole-body circulating BHB. In contrast to the modest phenotype in aged Hmgcl^S100a8-Cre^ mice, Hmgcl^LysM-Cre^ mice had no differences in body weight, glucose tolerance, or fasting-induced weight loss in 18 month-old male Cre-positive and Cre-negative littermate controls (Fig. 5, *B*–*D*).

**Figure 6:**
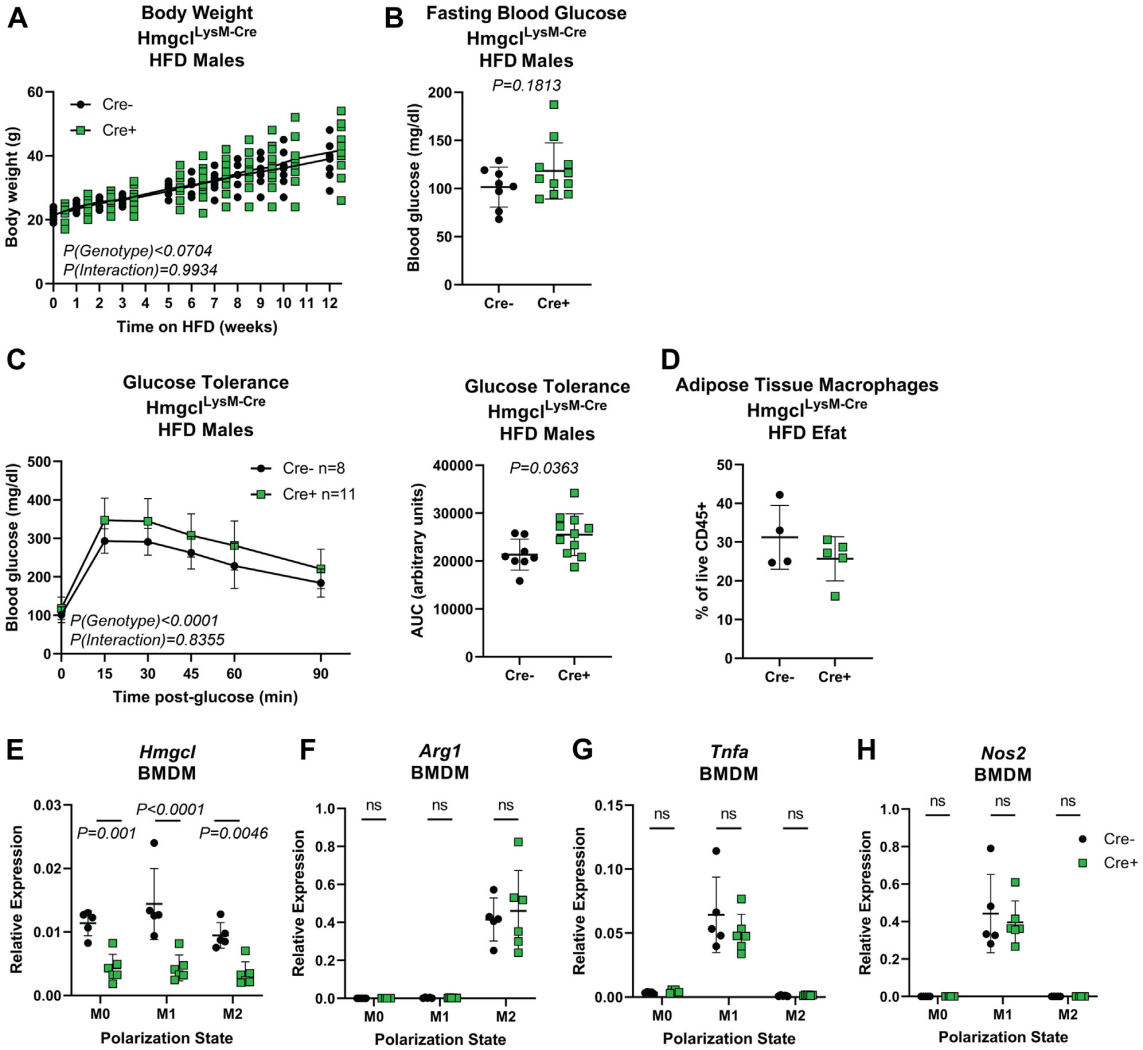
**Myeloid-specific *Hmgcl* expression modestly impacts metabolic phenotypes in high-fat diet-induced obese mice.** Male Hmgcl^LysM-Cre^ mice were fed a high-fat diet for 12 weeks to induce obesity. *A*, body weights were measured weekly throughout the experiment. Each symbol represents an individual mouse that was tracked over time and statistical differences were calculated by paired 2-way ANOVA. *Line* represents mean. *B*, 16-h fasting blood glucose was measured prior to glucose injection for glucose tolerance test. Each symbol represents an individual mouse and data are represented as mean ± SD. Statistical difference was calculated by *t* test. *C*, glucose tolerance was measured by ip glucose tolerance test (GTT, *left*) and total area under the curve was quantified (*right*). GTT Statistical differences were calculated by paired 2-way ANOVA and AUC differences were calculated by student’s *t* test. Data are represented as mean ± SD and each symbol in AUC panel represents an individual mouse. *D*, abundance of adipose tissue macrophages was determined by flow cytometry, defined as live CD45^+^CD11b^+^F4/80^+^. Each symbol represents a sample pooled from n = 2 mice. For (*E*–*H*), bone marrow–derived macrophages from Hmgcl^LysM-Cre^ mice were untreated (M0) or stimulated for 24 h with LPS+IFNγ (M1) or IL-4 (M2) to assess the role of HMGCL in macrophage polarization. Gene expression of (*E*) *Hmgcl*, (*F*) *Arg1*, (*G*) *Tnfa*, and (*H*) *Nos2* were measured by RT-PCR. Each symbol represents a BMDM sample that was generated by pooling bone marrow from n = 2 mice prior to differentiation. All data are expressed as mean ± SD and statistical differences were calculated by 2-way ANOVA to compare gene expression changes between genotypes within each polarization condition. BMDM, bone marrow–derived macrophage; HMGCL, 3-Hydroxy-3-Methylglutaryl-CoA Lyase; LPS, lipopolysaccharide.

Next, we tested if myeloid cell-intrinsic ketogenesis regulates obesity-induced inflammation. Male Hmgcl^LysM-Cre^ mice were fed a high-fat diet for 12 weeks to induce obesity. Despite no differences in body weight or fasting blood glucose, Cre-positive mice had lower glucose tolerance than Cre-negative littermates (Fig. 6, *A*–*C*). However, both genotypes had similar prevalence of adipose tissue CD11b+F4/80+ macrophages in their epididymal adipose tissue (Fig. 6*D*). Likewise, we measured no difference in M1/M2 polarization in Hmgcl-sufficient and Hmgcl-deficient bone marrow-derived macrophages (BMDMs) *in vitro* (Fig. 6, *E*–*H*). These data suggest the importance of ketogenesis in innate immune cells may depend on both cell type and physiological state.

## Discussion

Prior studies have conditionally ablated ketogenesis in extrahepatic tissues by targeting the pathway rate-limiting enzyme HMGCS2 (13, 41). However, this enzyme catalyzes the production of β-hydroxy-β-methylglutaryl CoA (HMG-CoA) from acetoacetyl CoA. HMG-CoA is then converted to acetoacetate by HMGCL. Therefore, possible confounding effects of loss of HMG-CoA cannot be ruled out. Notably, HMG-CoA can also be produced from leucine catabolism, so our approach blocks ketogenesis from this pathway as well. To focus exclusively on ketone body synthesis, we developed the *Hmgcl*^*fl/fl*^ mouse to ablate the terminal enzyme leading to production of all three ketone bodies, acetoacetate, BHB, and acetone. We validated the functional targeting of *Hmgcl* disruption in the Hmgcl^Alb-Cre^ mice by showing these mice fail to upregulate ketogenesis in response to KD and fasting. Our results in the Hmgcl^Alb-Cre^ mice also formally demonstrate that liver is the only organ that supports life-sustaining ketone body production during KD feeding and fasting and that all other combined sources of ketogenesis cannot compensate for the loss of hepatic ketogenesis.

The importance of ketone body production in nonhepatocytes is not completely understood. We and others have shown that immune cells are highly sensitive to ketone bodies through metabolic and nonmetabolic mechanisms (14, 19, 25, 26, 30, 31, 32, 33, 34, 35, 36). Moreover, the source of ketone bodies in immune modulation has not been defined and is likely different for each cell type and physiological condition. It is especially perplexing that short-lived neutrophils, with no obvious metabolic reliance on ketone bodies, would express ketogenic or ketolytic pathways. Given that innate immune cells express ketogenic enzymes and that their functions are impacted by both BHB and acetoacetate (19, 25, 26), we designed this study to define the role of neutrophil- and macrophage-intrinsic ketogenesis in regulating inflammation and metabolic health during aging. Because both BHB and acetoacetate have anti-inflammatory roles in macrophages, we expected the deletion of HMGCL in these cells to cause elevated inflammation that would exaggerate metabolic dysfunction in old mice. Surprisingly, we found only modest effects of conditional HMGCL ablation on physiological indicators of metabolic health in aged mice. In aged male neutrophil-specific Hmgcl^S100a8-Cre^ mice, we observed reproducible but small differences in body weights during aging, and this translated to corresponding slight improvement in glucose tolerance. We cannot rule out the possibility that Cre+ T and B cells, or Cre+ cells of unknown lineage in adipose tissue, albeit low in number, may be contributing to this phenotype (Fig. 3*B*). However, while adult Hmgcl^S100a8-Cre^ mice had lower inflammatory gene expression in response to monosodium urate crystal, aged Hmgcl^S100a8-Cre^ mice showed no change to acute inflammatory challenge with LPS. In contrast, mice with broader disruption of ketogenesis in all myeloid-lineage cells (Hmgcl^LysM-Cre^) did not replicate the aging phenotypes seen in Hmgcl^S100a8-Cre^ mice. These data raise the possibility that ketogenesis in different innate immune cell types may have different competing effects on whole-body physiology. Our data also suggest that exogenous ketone bodies from other organs are important for controlling age-related neutrophil and macrophage inflammation. Unfortunately, due to the >20% weight loss, that necessitates euthanasia of Hmgcl^Alb-Cre^ mice under ketogenic conditions, we were unable to directly test this possibility.

The results from our study still do not explain why innate immune cells express ketogenic enzymes. This is particularly intriguing in neutrophils, which are short-lived and highly glycolytic, and therefore have no obvious metabolic requirement for ketone bodies. Moreover, the lack of *Bdh1* expression in macrophages and neutrophils limits their potential metabolic ketone body utilization to acetoacetate, although this does not preclude a nonmetabolic role for exogenous BHB. Of note, prior literature examining ketone body utilization in innate immune cells has focused on BMDM, which do not reflect the diversity of macrophages *in vivo*. We suspect that the role of ketone pathways in innate immune cells may be cell type– and disease-specific. We studied this in the context of aging and obesity due to our prior work linking NLRP3 inflammasome activation to metabolic inflammation in these conditions (28, 29) and our subsequent discovery that BHB inhibits the NLRP3 inflammasome (25, 26). However, our *in vitro* data show that cell-intrinsic ketogenesis does not impact neutrophil NLRP3 activation or macrophage polarization. These data are in agreement with our prior work that acetoacetate does not inhibit NLRP3 inflammasome activation and that these cells likely do not produce their own BHB (25). These data probably also explain, at least in part, the modest phenotypes we observed *in vivo*. However, a limitation of the current study is that we only tested the role of *Hmgcl* gene deletion in a single mouse strain, C57BL6/J, and other strains may yield different results. Future studies using *in vivo* substrate tracing testing alternative sources of ketone bodies, and using additional disease models, should be carefully considered for testing the role of ketone pathways in immune cells.

## Experimental procedures

### Animals

All mice were housed in specific pathogen-free conditions under normal 12 h light/dark cycles. *Hmgcl*^fl/fl^ mice were generated by the Pennington Biomedical Research Center Transgenics Core. Genetic targeting to insert loxP sights into exon 2 of the *Hmgcl* allele was achieved by recombination of a PL253-loxP-frt-neo cassette in albino C57BL/6 embryonic stem cells. Upon confirmation of desired vector design and neomycin selection for initial enrichment of the targeted clone, we used these embryonic stem cells for injection into blastocysts for the generation of heterozygous Hmgcl-loxP–floxed mice. Using PCR to genotype these neo-founder mice, we then removed the neomycin-resistant drug marker by crossing to a recombinase FLP-derived mouse which recognizes the flippase recognition target for mediated cleavage and generation of our founder mice. These mice were crossed with C57BL6/J mice (Jax #000664) and then intercrossed to maintain a *Hmgcl*^*fl/fl*^ colony. *Hmgcl*^*fl/fl*^ mice were crossed to lineage-specific Cre-drivers (all on the C57BL6/J background) to generate conditional KO strains in liver (Albumin-Cre Jax #003574; (42)), neutrophils (S100a8-Cre Jax #021614; (43)), and myeloid cells (LysM-Cre Jax #004781; (44)). The majority of experiments were performed at Yale, and Hmgcl^fl/fl^ and Hmgcl^S100a8-Cre^ were sent to UCSF to breed mice for a portion of the neutrophil-specific experiments. For all comparisons, Cre-positive and Cre-negative *Hmgcl*^*fl/fl*^ littermates were used and genotypes were co-housed throughout lifespan until experimental endpoint to minimize effects of potential microbiome differences. All experimental procedures were performed with approvals from the Yale and UCSF Institutional Animal Care and Use Committee.

### Physiological measurements

Mice were housed in specific pathogen-free facilities and maintained on 12 h light/dark cycles. Standard and high-fat diets were irradiated for sterilization and all mice were provided ad libitum access to sterile drinking water in hydropacs. All tissue collections and physiological measurements were measured in the morning, within the first 5 h of the lights-on cycle, unless specifically described as otherwise. For KD experiments, mice were fed ad libitum KD for up to 1 week, as indicated in each figure (89.5% of calories from fat, 10.4% of calories from protein, 0.1% of calories from carbohydrates; Research Diets D19042606). For obesity experiments, male mice were fed a high-fat diet (60% of calories from fat; Research Diets D12492) for 12 weeks. For fasting experiments, mice were fasted for 24 h, beginning in the morning before tissue collection. For endotoxemia experiments, mice were challenged with LPS (O55:B5; 1 mg LPS/kg body weight) and euthanized 4 h later for analysis. Handheld meters were used to measure blood glucose (Contour Next) and BHB (Precision Xtra) levels in whole blood. For glucose tolerance tests, mice were fasted for 16 h prior to intraperitoneal injection of glucose (0.6 g glucose/kg body weight for old mice, 0.4 g glucose/kg body weight). Lipolysis was assessed by measuring glycerol release from adipose tissue explants (Sigma #MAK117). Body temperature was measured with a rectal temperature probe (BAT-12 microprobe thermometer, Physitemp).

### Neutrophil isolation

Primary neutrophils were isolated from the bone marrow of femurs of mice using the StemCell Technologies magnetic negative selection kit (Catalog # 19762) according to the manufacturer’s protocol. Femurs from at least n = 2 mice were pooled for neutrophil isolations, as we found the higher cell input improved enrichment purity. Purity was confirmed to be at least 95% (26). For NLRP3 activation, cells were treated with LPS (1 μg/ml, Sigma #L4391-1MG, strain 0111:B4) for 4 h, followed by 45 min ATP (5 mM, Sigma # A7699-1G).

### Bone marrow–derived macrophages

Mouse femurs were flushed, red blood cells were lysed with ACK lysis buffer, and remaining bulk bone marrow cells were cultured in RPMI (10% FBS + 1% antibiotic/antimycotic) in the presence of MCSF (20 ng/ml, Peprotech #315-02) for 7 days as described previously (25). For BMDM polarization, cells were counted and replated at 10^6^/ml in a 24-well plate. The following day, media was replaced with media (M0) or media containing LPS (1 μg/ml, O11:B4) + IFNγ (20 ng/ml, eBioscience #14-8311-63) for M1 polarization or IL-4 (10 ng/ml, eBioscience #14-8041-62) for M2 polarization. BMDM were cultured for 24 h in polarization media.

### Adipose tissue digestion

Gonadal white adipose tissue was minced and digested in 10 ml of digestion buffer for 45 min in a shaking (225 rpm) water bath. Digestion buffer: 1× HBSS supplemented with BSA (3% w/v, Sigma #A9647), Collagenase II (0.8 mg/ml, Worthington Biochemical #LS004174), calcium chloride (Sigma #21115, 1.2 mM), magnesium chloride (Sigma #M1028, 1 mM), and zinc chloride (Sigma #39059, 0.8 mM). After digestion, cells were centrifuged at 300*g* for 10 min and floating adipocyte fraction was discarded. The pellet containing the remaining stromal vascular fraction was washed at least twice with RPMI (5% FBS) over a 70 μm nylon filter, and red blood cells were lysed with ACK lysis buffer.

### Western blot

For all western blots, cell lysates were prepared in RIPA buffer containing protease and phosphatase inhibitors. Total protein concentrations were measured by BCA assay (Bio-Rad) and equal amounts of total protein were analyzed. Antibodies used were IL-1β (GeneTex # GTX74034), HMGCL (Proteintech # 16898-1-AP), Kbhb (PTM Biosciences #PTM-1201RM), and β-actin (Cell Signaling # 4967S).

### Flow cytometry

Cells were made into single-cell suspension by filtering over 70 μm filter. Red blood cells were lysed with ACK lysing buffer. Cells were incubated with Fc block and then stained with antibodies for standard lineage markers (CD3, B220, CD11b, Ly6G) for 30 min on ice, followed by three washes with FACS buffer, and then immediately acquired on a BD LSR II equipped with violet, red, green, and blue lasers, or an Attune NxT Flow Cytometer equipped with a blue, violet, green, and red laser. Data was analyzed with FlowJo. Antibodies were purchased from Biolegend.

### Gene expression

mRNA was isolated from cells in QIAzol using the Qiagen RNeasy kit. cDNA was transcribed using the iScript cDNA synthesis kit (Bio-Rad). Gene expression was measured by RT-PCR by ΔΔC_t_ method and expressed relative to 18s (Table S1).

### Statistical analyses

All graphs and statistical analyses were done in Prism (v9, GraphPad). For comparisons of two groups, two-sided student’s t-tests were used to calculate statistical differences. Comparisons of more than two groups were analyzed by 1-way ANOVA. To compare groups that were tracked over time, mice were individually tracked and statistical differences were calculated by paired 2-way ANOVA. *p*-values are provided in each figure, *p* > 0.05 was considered not significant (ns).

## Data availability

All data are contained within the article.

## Supporting information

This article contains supporting information.

## Conflict of interest

The authors declare they have no competing interests.
