# Supplementary material for: Innate immune cell-intrinsic ketogenesis is dispensable for organismal metabolism and age-related inflammation
Source: J Biol Chem. 2023 Feb 10;299(3):103005. doi: 10.1016/j.jbc.2023.103005 (PMC10025153; doi:10.1016/j.jbc.2023.103005)
Supplement: Supporting Table S1 and Figures S1, S2 [file mmc1.docx]

**Supporting Information for:**

**Innate immune cell-intrinsic ketogenesis is dispensable for organismal metabolism and age-related inflammation**

Emily L. Goldberg^1,^*, Anudari Letian^1^, Tamara Dlugos^2,3,4^, Claire Leveau^2,3,4^, Vishwa Deep Dixit^2,3,4,5^*

Included Material:

Table S1

Figure S1

Figure S2

| Gene Name (Symbol) | Forward Primer | Reverse Primer |
| --- | --- | --- |
| 3-Hydroxy-3-Methylglutaryl-CoA Lyase (*Hmgcl*) | GCTGATCGACATGCTTTCCG | GAGTGGTCAGCCATCTGTGG |
| Arginase 1 (*Arg1*) | ATTATCGGAGCGCCTTTCTC | TTTTTCCAGCAGACCAGCTT |
| Tumor Necrosis Factor (*Tnfa*) | AGGGTCTGGGCCATAGAACT | CCACCACGCTCTTCTGTCTAC |
| Nitric Oxide Synthase 2 (*Nos2*) | CCCTCCTGATCTTGTGTTGG | GGCAGTGCATACCACTTCAA |
| 18S rRNA (*18s*) | AACCCGTTGAACCCCATT | CCATCCAATCGGTAGTAGCG |

**Table S1. RT-PCR primer sequences used to measure gene expression**

**
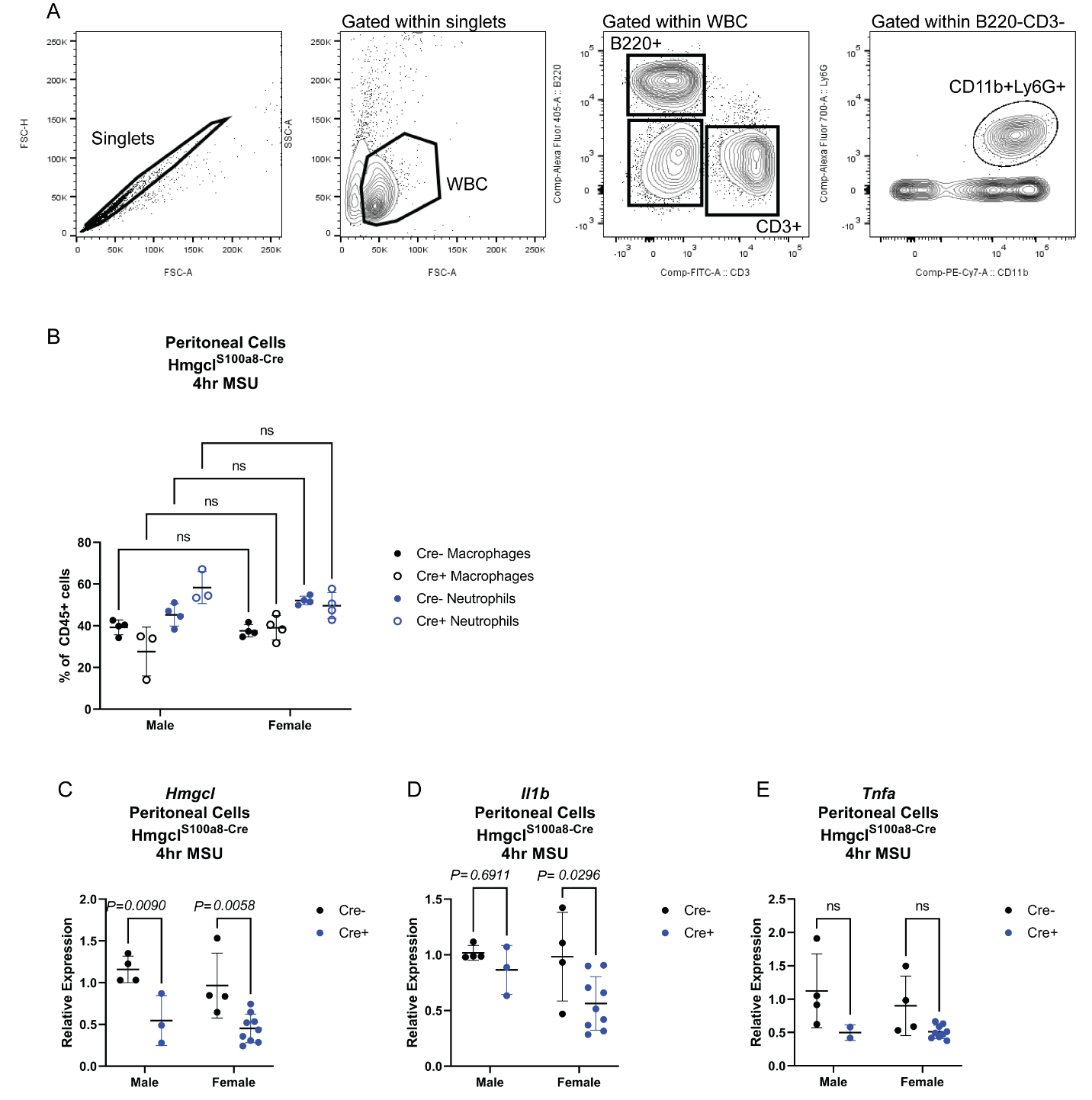
**

**Figure S1. Baseline characterization of neutrophil-specific HMGCL ablation.**

(A) Representative flow cytometry gating strategy to define B220+ B cells, CD3+ T cells, and CD11b+Ly6G+ neutrophils. For (B-E) These are the same data shown in Figure 3G-J, but with males and females separated. (B) Male and female macrophage and neutrophil abundance in the peritoneal cavity after ip injection of MSU. Gene expression of (C) *Hmgcl*, (D) *Il1b*, and (E) *Tnfa* was measured in whole peritoneal cell exudate 4 hours after MSU injection. For all graphs, each symbol represents an individual mouse. Data are represented as mean±SD and statistical differences of the indicated comparisons were calculated by 2-way ANOVA with Sidak’s correction for multiple comparisons (ns, not significant).

**
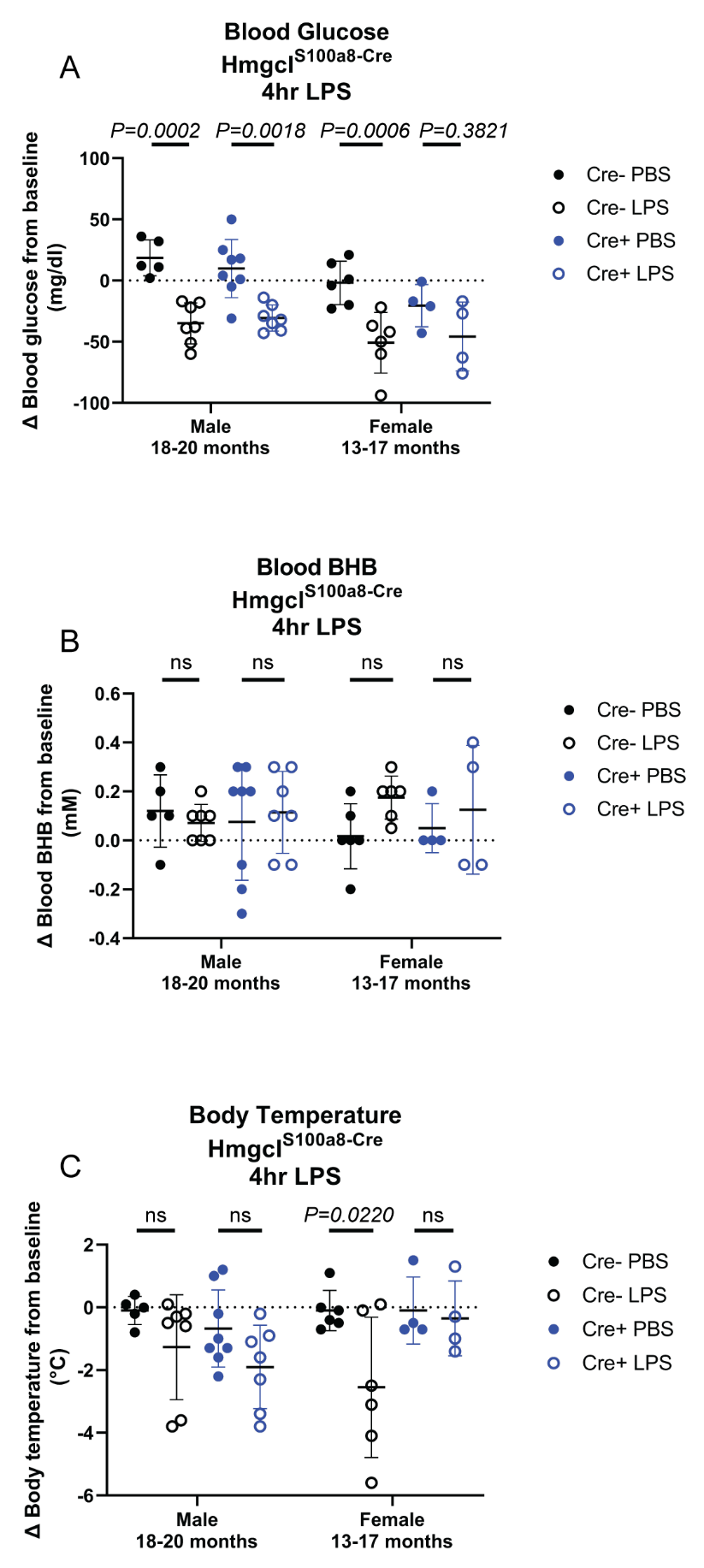
**

**Figure S2. Male and female physiological responses to LPS.**

These are the same data shown in Figure 4C-E, but with males and females separated. 18-20 month-old males and 13-17 month-old females were injected with LPS or PBS control and changes in (A) blood glucose, (B) blood BHB, and (C) body temperature were measured 4 hours later to assess the physiological response to acute inflammation. Statistical differences in for all graphs were calculated by 2-way ANOVA with Sidak’s correction for multiple comparisons within each sex (ns: not significant). For all graphs, each symbol represents an individual mouse and all data are expressed as mean±SD.
